# Clinical outcomes and safety of passive leg raising in out-of-hospital cardiac arrest: a randomized controlled trial

**DOI:** 10.1186/s13054-021-03593-7

**Published:** 2021-05-25

**Authors:** Youcef Azeli, Alfredo Bardají, Eneko Barbería, Vanesa Lopez-Madrid, Jordi Bladé-Creixenti, Laura Fernández-Sender, Gil Bonet, Elena Rica, Susana Álvarez, Alberto Fernández, Christer Axelsson, Maria F. Jiménez-Herrera

**Affiliations:** 1 Sistema d’Emergències Mèdiques de Catalunya, Carrer de Pablo Iglesias 101–115, L’Hospitalet de Llobregat, Barcelona, Spain; 2 Emergency Department, Sant Joan University Hospital, Reus, Spain; 3 Institut d’Investigació Sanitària Pere Virgili (IISPV), Reus, Spain; 4 Cardiology Department, Joan XXIII, University Hospital, Tarragona, Spain; 5 Universitat Rovira i Virgili, Tarragona, Spain; 6 Pathology Service, Institute of Legal Medicine and Forensic Sciences of Catalonia, Tarragona, Spain; 7 Atenció Primaria, Institut Català de la Salut, Tarragona, Spain; 8 Llevant Clinic Unit, Santa Tecla Hospital, Tarragona, Spain; 9 Department de Enginyeria Informàtica i Matemàtiques, Universitat Rovira i Virgili, Tarragona, Spain; 10 Departament d’Enginyeria Química, Universitat Rovira i Virgili, Tarragona, Spain; 11 Center of Prehospital Research, Faculty of Caring Science, Work Life and Social Welfare, University of Borås, Borås, Sweden; 12 Department of Nursing, Universitat Rovira i Virgili, Tarragona, Spain

**Keywords:** Cardiopulmonary resuscitation, Passive leg raising, Adverse effect, Out-of-hospital cardiac arrest

## Abstract

**Background:**

There are data suggesting that passive leg raising (PLR) improves hemodynamics during cardiopulmonary resuscitation (CPR). This trial aimed to determine the effectiveness and safety of PLR during CPR in out-of-hospital cardiac arrest (OHCA).

**Methods:**

We conducted a randomized controlled trial with blinded assessment of the outcomes that assigned adults OHCA to be treated with PLR or in the flat position. The trial was conducted in the Camp de Tarragona region. The main end point was survival to hospital discharge with good neurological outcome defined as cerebral performance category (CPC 1–2). To study possible adverse effects, we assessed the presence of pulmonary complications on the first chest X-rays, brain edema on the computerized tomography (CT) in survivors and brain and lungs weights from autopsies in non-survivors.

**Results:**

In total, 588 randomized cases were included, 301 were treated with PLR and 287 were controls. Overall, 67.8% were men and the median age was 72 (IQR 60–82) years. At hospital discharge, 3.3% in the PLR group and 3.5% in the control group were alive with CPC 1–2 (OR 0.9; 95% CI 0.4–2.3, *p* = 0.91). No significant differences in survival at hospital admission were found in all patients (OR 1.0; 95% CI 0.7–1.6, *p* = 0.95) and among patients with an initial shockable rhythm (OR 1.7; 95% CI 0.8–3.4, *p* = 0.15). There were no differences in pulmonary complication rates in chest X-rays [7 (25.9%) vs 5 (17.9%), *p* = 0.47] and brain edema on CT [5 (29.4%) vs 10 (32.6%), *p* = 0.84]. There were no differences in lung weight [1223 mg (IQR 909–1500) vs 1239 mg (IQR 900–1507), *p* = 0.82] or brain weight [1352 mg (IQR 1227–1457) vs 1380 mg (IQR 1255–1470), *p* = 0.43] among the 106 autopsies performed.

**Conclusion:**

In this trial, PLR during CPR did not improve survival to hospital discharge with CPC 1–2. No evidence of adverse effects has been found.

*Clinical trial registration*

ClinicalTrials.gov: NCT01952197, registration date: September 27, 2013, https://clinicaltrials.gov/ct2/show/NCT01952197.

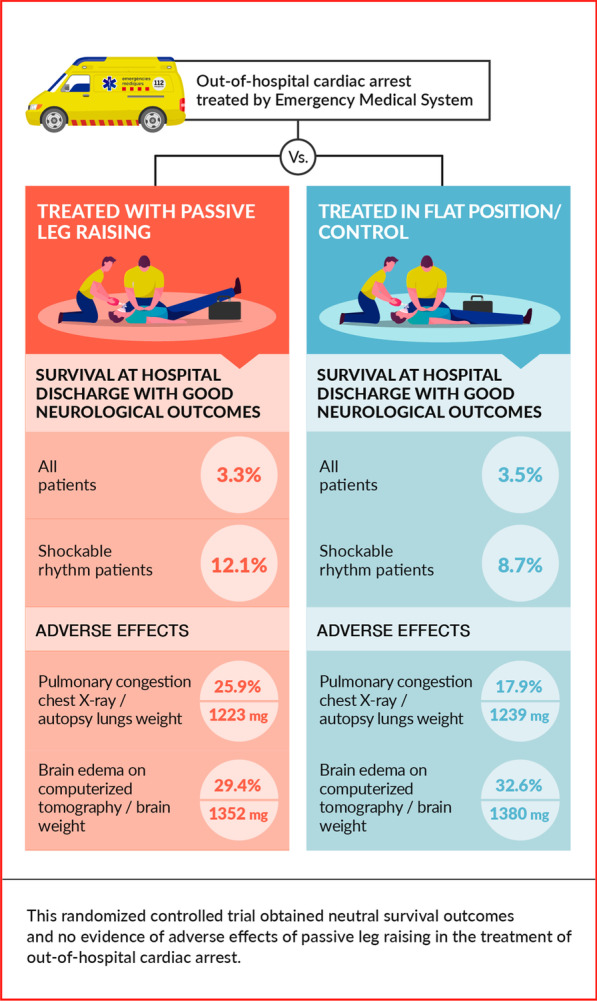

## Introduction

Despite the efforts made in the last 2 decades, survival of out-of-hospital cardiac arrest (OHCA) hardly reaches 10% [1, 2]. Survival is determined by several factors such as the performance of early bystander cardiopulmonary resuscitation (CPR), the use of public automatic external defibrillators (AEDs), the performance of high-quality CPR or the post-resuscitation care provided in the hospital [3]. Performing chest compressions at an appropriate depth, at a frequency of between 100 and 120 pm, while ensuring the return of the sternum to its original position during decompression, are key elements of quality CPR that aim to optimize cardiac output during CPR [4].

In early CPR guidelines, passive leg raising (PLR) was considered to be a maneuver that could promote venous return and increase artificial circulation during chest compressions [5]. In 1992, this statement was removed due to lack of evidence [6]. PLR mimics rapid volume expansion and is often used in intensive care units during the hemodynamic assessment of patients [7]. During CPR, cardiac output is limited, leading to a low flow state [8, 9]. Increasing the venous and arterial bed resistances can improve myocardial and cerebral blood flow [10]. PLR stresses the volume of the venous reservoir, increasing the mean systemic filling pressure, which is the driving pressure of the venous return flow [11]. The coronary perfusion pressure (CPP) is a good predictor of the return of spontaneous circulation (ROSC) [12]. In a series of resuscitated pigs, PLR increased coronary perfusion pressure and improved neurological outcomes compared to a control group. A retrograde volume loading of the aorta from the PLR may occur, raising the intra-abdominal pressure and the anterograde blood flow resistance, which increases the CPP gradient [13]. A study of OHCA showed that 20º of leg elevation helped to increase cardiac output during CPR [14]. Despite these promising hemodynamic results, a recent observational study introducing PLR into the standard treatment of patients with OHCA found no difference in survival compared to a control group [15].

In recent years, there has been growing concern about the safety of various interventions performed by emergency teams during resuscitation. Fluid infusion during resuscitation has led to worsen clinical outcomes [16, 17]. Another CPR body position, such as Trendelenburg, was associated with an increased intracranial pressure [18]. There are no data about the safety of PLR during CPR, and the beneficial effect of PLR performed during CPR is still unknown. We hypothesized that PLR performed at the beginning of OHCA treatment by a medical emergency system will be a safe maneuver and will improve survival at discharge with good neurological outcomes compared to patients treated in a standard way.

## Methods

### Trial design and setting

This is a randomized controlled trial with blinded assessment of the outcome (ClinicalTrials.gov Identifier: NCT01952197). This study was conducted by the Emergency Medical System (EMS) of Catalonia in the region of Camp de Tarragona. It is the only EMS in the Camp de Tarragona region and provides assistance to 100% of the territory. This study region has an area of 2704.3 km^2^ and 511,622 inhabitants. The population density varies between urban and rural areas and was mainly distributed close to the coast. The mean density in 2014 was 190.7 hab/km^2^. The two main municipalities form together the second largest metropolitan area in Catalonia. A multiple information source prospective registry for the study of sudden death and adverse effects of CPR was conducted (ReCaPTa Study) during the study period [19].

When this study began, there were two types of ambulances regularly distributed in the territory: 42 basic life support (BLS) staffed by two healthcare technicians and 4 advanced life support (ALS) staffed by one physician, one nurse and one healthcare technician. The study has the ethical approval of the Ethical Research Committee in Tarragona (15/2013) and Reus (13-04-25/4aclaobs1). A waiver of informed consent was obtained. The study was conducted in accordance with the Declaration of Helsinki and Good Clinical Practices.

### Intervention and randomization

We included all patients who presented an OHCA in which the EMS performed a CPR attempt between April 2014 and April 2017. Patients under 18 years of age were excluded. We also excluded patients whose pathology or previous condition made PLR contraindicated or unfeasible according to the treating physician's criteria such as traumatic patients with suspected pelvic or lower limb fracture or pregnant women.

When a cardiac arrest is suspected, the dispatch center activates two ambulances, a BLS, which usually arrives first, equipped with an AED, and an ALS.

Enrolment was performed on the scene at the initial cardiac arrest assessment. Manual CPR was started, and if there were no exclusion criteria, randomization and allocation concealment was performed via an opaque and sealed envelope system. The randomization process and the description of the protocol are detailed in the published study design [20]. PLR was performed within the first 5 min after the arrival of the first ambulance and was maintained until the end of CPR or until the patient presented ROSC. The angle of PLR was set between 20° and 45° following previous data [10]. To ensure that the legs were lifted at this angle different assays were made, so all ambulances were equipped with a 20-cm-high stool and it was recommended that one of the bags of the resuscitation equipment be placed between the stool and the patient's legs.

Prior to the start of the study, all staff were provided with training sessions, study protocol documentation which included photographs on how the intervention was to be performed and an instructional video. The study was promoted on social networks, and the percentage of cases enrolled in the study was monitored.

The sample of the study initially estimated was 1490 patients in each group and it was calculated for an incidence of 40/100,000 inhabitants and for an increase of survival to discharge from 7 to 10%. The power was set at 80% and a bilateral two-tailed significance of 5%. The plan was to include 300 patients in each group during the first 3 years of the study in the whole district of Tarragona and 188 patient in each group for the region of Camp de Tarragona [20].

### Data collection and quality control

The resuscitation-related data were prospectively collected by the medical crew after attending the OHCA following the Utstein style using an online application available in the computer system of each base or in the personal mobile devices [21]. The data collected were: date of the alarm, age, sex, reasons for not attempting resuscitation, non-randomization reasons, performance of passive leg raising, randomization number, first monitored rhythm, witnessed status, type of first ambulance to provide assistance, bystander CPR before ambulance arrival, cardiac arrest location, presumed cardiac arrest etiology, treatment provided including mechanical chest compression, intubation, drugs such as adrenaline and amiodarone and defibrillation, and number of defibrillations. The times of cardiac arrest, call, first defibrillation and arrival of EMS are based on the times automatically collected by the coordination center. The shock from an AED used by a bystander or by a BLS prior to the arrival of the ALS was recorded as a shockable rhythm (ventricular fibrillation/pulseless ventricular tachycardia). This study did not collect information from public AEDs. The sensitivity and specificity of an AED shock is high, making it difficult to over-diagnose shockable rhythms [22, 23]. The initial ETCO2 measurement was collected immediately after orotracheal intubation [24].

The database obtained was subjected to an exhaustive quality control by trained personnel who reviewed all the case reports generated by the dispatch center. Case reports coded with the all used CIE.9 code related to the OHCA were reviewed (798.1 (instantaneous death), 798.9 (Unattended death), 427.5 (cardiac arrest) and 427.41 (ventricular fibrillation). All of the BLS manual paper records in which resuscitation was initiated were also collected and reviewed. The missing information was completed through medical reports or requests to the medical crews who attended the case.

Survivors were followed by hospital and primary care investigators who did not have access to the intervention performed. An interview was performed prior to hospital discharge, and a detailed review of the medical chart was performed at 1 year. The neurological assessment of the survivors was performed using the Pittsburgh cerebral performance category (CPC) at discharge and at 1 year. CPC 1 indicates no disability, CPC 2 slight disability, CPC 3 moderate disability, CPC 4 comatose/vegetative state and CPC 5 death.

For the assessment of post-resuscitation pulmonary complications, the report of the attending physician or radiologist on the first X-ray taken upon arrival at the hospital was evaluated [8]. Lung complications were considered when bilateral lung opacities, edema, pulmonary congestion or bilateral alveolar pattern was described. The first head computerized tomography (CT) radiologist report was taken into account to evaluate the brain edema in survivors. Where required by law, non-survivors were studied by autopsy following the protocol of the Institute of Legal and Forensic Medicine of Catalonia, which is focused on the study of sudden death and the adverse effects of CPR [25]. Lung and brain weight at autopsy is routinely collected as a part of the sudden death protocol study and is a good indicator of the extravascular lung water found in the pulmonary and brain edema [26, 27]. Autopsies were performed by a forensic team specialized in the study of the causes of sudden death blinded to the intervention studied within the first 24 h after death.

### Outcomes

The primary end point was survival to hospital discharge with good neurological outcomes (CPC 1–2). The secondary end points were the initial ETCO2; survival at hospital admission; survival at hospital discharge with good neurological outcomes (CPC 1–2) in all patients and in patients with initial shockable rhythm; survival at 1 year with good neurological outcomes (CPC 1–2) in all patients and in patients with shockable rhythm; pulmonary complications on the first chest radiography at the hospital; brain edema on the CT and lung, and brain weight from autopsies.

### Statistical analyses

The continuous variables were described with median and interquartile ranges and the categorical ones with number of cases and percentages. The Student’s *T* or Mann–Whitney’s *U* and the Chi-square were used to compare the subgroups.

The primary survival outcomes analysis was performed in the intention-to-treat population, which included patients randomized to the intervention assigned confirmed and treated by the EMS. The end point variables in this study were categorical, and data were presented in proportions, percentages and 95% confidence interval (CI). To find out the possible differences between patients of the subgroups, Pearson *χ*2 tests for comparison of proportions were conducted, and odds ratios with their 95% CIs were calculated. To control the type I error rate in our clinical trial, we used interim monitoring by O'Brien–Fleming frequentist method [28]. We performed interim statistical analyses once a year, in April. The alpha spending function approach was used as previously described [29]. Given the neutral result obtained in the interim analysis and the difficulties in following up the survivors in other study areas, the steering committee of the study interrupted the recruitment of patients.

All tests were two-tailed and *p* values below 0.05 were considered statistically significant. All the statistical analyses were performed using R software version 4.0.0.

## Results

**Fig. 1:**
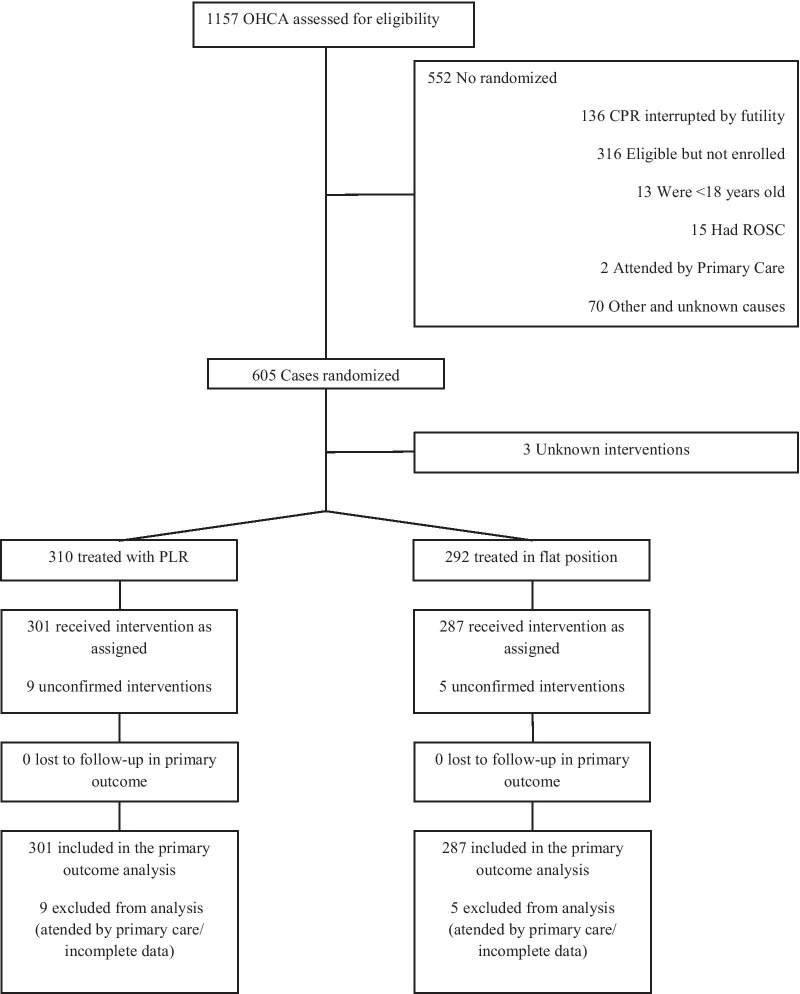
Trial flowchart

During the study period, BLS was initiated in 1157 patients who presented an OHCA and were assessed for eligibility. The enrolment, the allocation process and the follow-up are shown in Fig. 1. On 1157 patients assessed for eligibility, 605 were randomized. After exclusions, 588 OHCAs were included in the intention-to-treat survival analysis out of which 301 (51.2%) were treated with PLR and 287 (48.8%) were treated in flat position. In 143 patients, CPR maneuvers were interrupted on arrival of the ALS physician due to futility. The main causes of futility were medical background (56.6%), advanced age (45.4%) and injuries incompatible with life (5.6%). Among the 445 resuscitation attempts, 234 (52.5%) were treated with PLR and 211 (47.4%) were controls.

The characteristics of the study population and the comparison of Utstein variables are shown in Table 1. The initial ETCO2 measurement was recorded in 207 of 374 intubated patients. The median of the initial ETCO2 in the PLR group was 28 mmHg (IQR 14–48) and 27 mmHg (IQR 17–45) in the control group (*p* = 0.99). The hospital variables and outcomes according to the intervention performed are shown in Table 2.

**Table 1:** Characteristics of the study population

	Passive leg raising (*n* = 234)	Control (*n* = 211)	Absolute difference (95%CI), %
Age (years)	70 (59–80)	69 (57–77)	1.0 (0.0 to 5.0)
Sex (female)	75 (32.1)	52 (24.6)	7.4 (− 0.9 to 15.7)
Location of cardiac arrest			
Home	169 (72.2)	141 (66.8)	5.4 (− 3.2 to 14.0)
Public place	55 (23.5)	63 (29.9)	5.52 (− 2.2 to 13.2)
Witnessed status			
Bystander witnessed	171 (73.4)	148 (70.8)	2.6 (− 6.0 to 10.9)
Crew witnessed	19 (8.1)	11 (5.2)	2.9 (− 1.7 to 7.5)
Bystander CPR	86 (37.1)	80 (37.9)	− 0.8 (− 9.9 to 8.2)
Initial assistance by BLS	119 (71.7)	102 (68.9)	2.8 (− 7.4 to 12.9)
Initial rhythm			
Shockable rhythm	63 (26.9)	64 (30.5)	− 3.5 (− 12.0 to 4.9)
PEA	4 (1.7)	8 (3.8)	− 2.1 (− 5.2 to 0.1)
Asystole	167 (71.4)	138 (65.7)	5.6 (− 3.0 to 14.3)
Treatment			
Adrenalin	220 (94.0)	195 (92.4)	1.6 (− 3.1 to 6.3)
Intubation	192 (82.1)	182 (86.7)	− 4.6 (− 11.3 to 2.1)
Amiodarone	40 (17.1)	38 (18.0)	− 0.9 (− 8.0 to 6.2)
Defibrillation^a^	87 (37.2)	92 (43.6)	− 6.4 (− 15.5 to 2.7)
Number of defibrillation	2 (1–4)	3 (2–5)	− 0.6 (− 1.7 to 0.5)
Mechanical chest compressions	40 (17.1)	37 (17.5)	− 0.4 (− 7.5 to 6.6)
Initial ETCO2, mmHg			
Initial ETCO2 in all patients	28 (14–48)	27 (17–45)	0.2 (− 5.7 to 6.0)
Initial ETCO2 in patients with shockable rhythm^b^	30 (22–53.5)	26 (17.75–48)	4.0 (− 5.0 to 14.0)
Presumed cardiac arrest etiology			
Cardiac	179 (76.5)	144 (68.6)	7.9 (− 0.4 to 16.2)
Toxics	1 (0.4)	3 (1.4)	− 1.0 (− 2.8 to 0.8)
Traumatic	5 (2.1)	8 (3.8)	− 1.7 (− 4.9 to 1.5)
Respiratory	13 (5.6)	11 (5.2)	0.3 (− 3.9 to 4.5)
Neurologic	1 (0.4)	0 (0.0)	0.4 (− 0.4 to 1.2)
Drowning	6 (2.6)	11 (5.2)	− 2.7 (− 6.3 to 1.0)
Pulmonary embolism	1 (0.4)	2 (1.0)	− 0.5 (− 2.1 to 1.0)
Others	28 (12.0)	31 (14.8)	− 2.8 (− 9.1 to 3.5)
Delay			
Collapse to call EMS, min	5 (2–11)	4 (2–6)	1.3 (− 0.2 to 3.0)
Collapse to start CPR, min	6 (2–11)	8 (3–11)	− 2.0 (− 2.0 to 1.0)
Collapse to first defibrillation, min	14 (8–28)	12 (8–21)	1.5 (− 2.0 to 5.0)
Call to EMS to EMS arrival, min	10 (7–13)	9 (7–11)	0.6 (− 0.3 to 1.5)

Values are *n* (%) and median (interquartile range)

*EMS* emergency medical system, *BLS* basic life support ambulance, *PEA* pulseless electrical activity, *CPR* cardiopulmonary resuscitation, *ETCO2* end tidal CO_2_

^a^Number of patients who received defibrillation in any moment during CPR

^b^Only patients with initial shockable rhythm were included

**Table 2:** Hospital data

	Passive leg raising (*n* = 234)	Control (*n* = 211)	*p* Value
Return of spontaneous circulation	65 (27.8)	57 (27.0)	0.86
Transport to the hospital	59 (25.2)	57 (27.0)	0.67
Survival at hospital admission	52 (22.2)	49 (23.2)	0.80
Variables at admission			
Initial pH	7.17 (6.99–7.46)	7.18 (7.09–7.28)	0.79
Lactate	4.5 (3.0–7.5)	5.0 (4.0–6.9)	0.79
Mean arterial pressure, mmHg	81 (70–93)	78 (65–86)	0.50
Radiologic findings			
Pulmonary complications on chest X-rays	7 (25.9)	5 (17.9)	0.47
Brain edema on computerized tomography	5 (29.4)	10 (32.6)	0.84
Post-resuscitation care^a^			
Days in intensive care unit	2.0 (1.0–5.2)	5 (3–7)	0.41
Percutaneous cardiac intervention	7 (13.5)	6 (12.2)	0.86
Controlled hypothermia treatment	10 (19.2)	12 (24.5)	0.52
Vasoactive drugs	17 (32.7)	22 (44.9)	0.21
Seizures activity	8 (28.6)	11 (30.6)	0.86
Hospital cause of death			
Cardiac	26 (57.8)	22 (53.7)	0.70
Respiratory	5 (11.1)	6 (14.6)	0.63
Neurologic	3 (6.7)	4 (9.8)	0.60
Drowning	4 (8.9)	3 (7.3)	0.79
Traumatic	2 (4.4)	2 (4.9)	0.92
Pulmonary embolism	2 (4.4)	1 (2.4)	0.61
Toxics	0	2 (4.9)	0.13
Others	3 (6.7)	1 (2.4)	0.35
Survival at hospital discharge	15 (6.4)	14 (6.6)	0.92
Survival at 1 year	10 (4.3)	11 (5.3)	0.63

Values are *n* (%) or median (Interquartile range)

^a^Calculated ratio among all the survivors at hospital admission

### Safety data

Regarding adverse effects, the incidence of pulmonary complications in the first chest X-rays and brain edema on the CT were similar in the PLR group and the control group, 25.9% versus 17.9% (*p* = 0.47) and 29.4% versus 32.6% (*p* = 0.84) respectively. Among the 445 resuscitation attempts, 106 were studied by autopsy. The autopsy findings including the cause of death and anthropometric variables are shown in Table 3. There was no difference in lung and brain weight collected in the autopsy study 1223 mg (IQR 909–1500) and 1352 mg (IQR 1227–1457) in the PLR group versus 1239 mg (IQR 900–1507) and 1380 mg (IQR 1255–1470) in the control group (*p* = 0.82 and *p* = 0.43, respectively). No other adverse effects were reported.

**Table 3:** Autopsy findings

	Passive leg raising (*n* = 55)	Control (*n* = 51)	*p* Value
Autopsy causes of death			
Cardiac	29 (56.9)	29 (59.2)	0.81
Drowning	3 (5.9)	6 (12.2)	0.27
Pulmonary embolism	3 (5.9)	6 (12.2)	0.27
Toxics	2 (3.9)	4 (8.2)	0.37
Traumatic	2 (3.9)	1 (2.0)	0.58
Respiratory	3 (5.9)	0	0.08
Neurologic	2 (3.9)	1 (2.0)	0.58
Vascular	3 (5.9)	0	0.08
Digestive	2 (3.9)	1 (2.0)	0.58
Others	2 (3.9)	1 (2.0)	0.58
Anthropometric variables			
Weight, kg	80 (72–95)	86 (70–96)	0.43
Height, m	1.65 (1.61–1.71)	1.67 (1.61–1.74)	0.38
Body Mass Index, kg/m^2^	30.12 (26.4–33.4)	30.4 (27.2–33.6)	0.81
Intrathoracic visceral findings			
Heart weight, mg	457 (394–600)	500 (410–578)	0.51
Lung weight, mg	1223 (909–1500)	1239 (900–1507)	0.82
Brain weight, mg	1352 (1227–1457)	1380 (1255–1470)	0.43

Values are *n* (%) or median (Interquartile range)

### Clinical outcomes data

The detail of the survival outcomes analysis among all patients and patients with shockable rhythm are shown in Table 4. The number of patients who survived at hospital discharge with good neurological outcome (CPC 1–2) was 10 of 331 (3.3%) in the PLR group versus 10 of 287 (3.5%) in the control group (OR: 0.9; 95% CI 0.4–2.3, *p* = 0.91). No significant differences in survival at hospital admission were found in all patients (OR 1.0; 95% CI 0.7–1.6, *p* = 0.95) and among patients with an initial shockable rhythm (OR 1.7; 95% CI 0.8–3.4, *p* = 0.15).

**Table 4:** Survival outcomes

	Passive leg raising	Control	Odds ratio (95% CI)	*p* Value
Survival at hospital admission				
All patients	52/301 (17.3)	49/287 (17.1)	1.0 (0.7–1.6)	0.95
Patients with shockable rhythm	28/66 (42.4)	21/69 (30.4)	1.7 (0.8–3.4)	0.15
Survival at hospital discharge CPC 1–2				
All patients	10/301 (3.3)	10/287 (3.5)	0.9 (0.4–2.3)	0.91
Patients with shockable rhythm	8/66 (12.1)	6/69 (8.7)	1.4 (0.5–4.4)	0.51
Survival at 1 year with CPC 1–2				
All patients	9/299 (3.0)	8/284 (2.8)	1.1 (0.4–2.8)	0.89
Patients with shockable rhythm	8/66 (12.1)	5/67 (7.5)	1.7 (0.5–5.5)	0.37

Values are *n* of patients with outcome/total *n* patients (%)

*CPC* cerebral performance category, *CI* confidence interval, *CPC* cerebral performance category

## Discussion

The results of this randomized controlled trial studying the effectiveness of PLR in the treatment of OHCA reveal no differences between the PLR group and the control group with regard survival to hospital discharge with good neurological outcome.

The baseline characteristics of each group are comparable. The survival results in our study are in the same line as those obtained by an observational study that introduced PLR in the treatment of OHCA and compared its effectiveness with a matched group that received standard treatment. In this Swedish study, PLR was performed more often in cases with a worse clinical scenario and it was suggested that early leg elevation could improve its benefit on survival [15].

According to the results obtained, PLR during CPR is a safe intervention. The pulmonary complications rate observed in the first chest X-rays and the incidence of brain edema on CT were similar to that of other studies [30, 31]. The autopsy study provides objective data on lung congestion and brain edema. Approximately 1/3 of non-survivors underwent an autopsy, which is similar to or even higher than other safety OHCA studies [32, 33].

The idea of an transient effect of PLR over time has been described in cases of septic patients and is attributed to capillary leak [34]*.* During cardiac arrest, maintained hypoxia has a similar effect on capillary permeability, which could favor the shortened effect of PLR on systemic mean filling pressure (Pmsf) and cardiac output [35]. In any case, optimizing Pmsf and venous return is key to improving survival outcomes. A personalized physiology-guided resuscitation protocol recently published considers increase the circulatory volume in patients with pre-arrest CVP < 2 mmHg using PLR [36].

Experimental data support the distinct hemodynamic effect of PLR and volume load during CPR. Volume loading has been associated with a decrease in CPP due to the detrimental effect of the increase in right atrial pressure (RAP) in the decompression phase [37]. However, PLR seems not to alter RAP and has been associated with an increase in CPP which is key to obtain ROSC [13]. On the other hand, our results regarding neurologic outcomes support that PLR does not increase intracranial pressure as may occur in other body positions. The effect of gravity may impair venous drainage from the brain to the heart and increase intracranial pressure in the case of CPR in Trendelenburg position [38].

A higher but no significant survival rate at hospital admission was found in our study in favor of PLR among patients with a shockable rhythm, suggesting that PLR may be useful in optimizing the conditions prior to an attempt of defibrillation during hemodynamically guided CPR [39, 40]. It should be considered that the greatest change in cardiac output due to PLR occurs after 1 min of the procedure [14]. On the other hand, PLR could trigger the Bainbridge reflex and might help to restart the electrical activity after ventricular fibrillation termination [41].

The ETCO2 concentration is a reliable marker for monitoring cardiac output and, therefore, CPP during CPR and it is used as a prognostic factor [42, 43]. The initial ETCO2 < 10 mmHg has been proposed as a predictor of bad outcome in OHCA [44]. No differences have been found in the initial ETCO2 between the PLR group and the control group. The effect of PLR on cardiac output and ETCO2 does not appear to be maintained beyond the first 4 min [14]. A similar shortened increase in CPP over time attributed to a rise of diastolic aortic pressure was described in an animal model [13]. This could justify our findings due to a longer delay between PLR at the BLS arrival and intubation during the ALS assistance.

Regarding new study designs, it would be helpful to keep in mind the time of the maximum hemodynamic effect of PLR. Measuring the effectiveness on survival outcomes of PLR performed 1 min prior to a first or a second defibrillation attempt may be the basis for new studies. Further investigations are warranted to establish the utility of this simple maneuver in the setting of a cardiopulmonary resuscitation hemodynamically guided or in the treatment of refractory ventricular fibrillation.

### Strength and limitations

The main strength of this research is a robust randomization process as a consequence of the easy performance of the studied intervention. In very few patients, the maneuver was considered contraindicated by medical criteria. It only occurred in one case with a lower limb amputation or two cases of traumatic shock in which an unstable pelvis or lower limb fracture was suspected. There was little loss of cases in the follow-up of the survivors and in the main outcome variables. The safety study included the objective data from autopsies that were performed on a high proportion of the patients. Therefore, the safety study involved both survivors and non-survivors, allowing us to assess the risk–benefit of PLR.

The difference between the samples obtained and the required samples set out in the design probably resulted in a lack of power to detect significant differences in the clinical outcomes, which is the main limitation. The low rate of bystander CPR found in our study which can lead to a low survival rate may have also contributed to neutral survival results. Therefore, the results of this first randomized trial in this topic should be interpreted with care.

The angle of leg elevation performed in each case was not measured and we cannot rule out variability in how the intervention was performed. The time between the cardiac arrest and the PLR was not collected which could be a potential confounder. During this study, some patients received CPR during transport to the hospital when presenting a re-arrest or when being enrolled in an ongoing CPR protocol with direct transfer to the cath laboratory. In these cases, we cannot guarantee that PLR was maintained during the entire period of CPR. Lung and brain weights as a safety outcome were only measured in autopsied patients. Only a few autopsies were from survivors, but it is possible that some may have been treated for lung edema in the hospital prior to death.

## Conclusion

Passive leg raising in the treatment of OHCA did not improve survival at hospital admission or discharge with good neurological outcomes in this randomized controlled trial. No differences were found in the incidence of pulmonary complications or brain edema between the group treated with passive leg raising and the control group. No other adverse effects were reported, indicating that PLR during CPR is a safe intervention.

## Data Availability

The datasets during and/or analyzed during the current study are available from the corresponding author on reasonable request.

## Abbreviations

AED: Automatic external defibrillator
ALS: Advance life support
BLS: Basic life support
CI: Confidence interval
CPC: Cerebral performance category
CPP: Coronary perfusion pressure
CPR: Cardiopulmonary resuscitation
CT: Computerized tomography
ETCO2: End tidal CO_2_
IQR: Interquartile range
EMS: Emergency medical system
OHCA: Out-of-hospital cardiac arrest
PLR: Passive leg raising
Pmsf: Mean systemic filling pressure
RAP: Right atrial pressure
ROSC: Return of spontaneous circulation
